# A Quality Improvement Project to Increase Mother’s Milk Use in an Inner-City NICU

**DOI:** 10.1097/pq9.0000000000000204

**Published:** 2019-08-30

**Authors:** Nikita S. Kalluri, Laura A. Burnham, Adriana M. Lopera, Donna M. Stickney, Ginny L. Combs, Bernadette M. Levesque, Barbara L. Philipp, Margaret G. Parker

**Affiliations:** From the * Department of Pediatrics, Boston Medical Center, Boston, Mass.; † Boston University School of Medicine, Boston, MA.

## Abstract

Mother’s milk is recommended for preterm infants due to numerous health benefits. At our inner-city hospital, >80% of mothers of infants younger than 34 weeks’ gestation initiated milk production, but fewer continued until discharge. Among infants younger than 34 weeks’ gestation, we aimed to (1) increase any mother’s milk use in the 24 hours before discharge/transfer to *>*75%; (2) increase exclusive mother’s milk use in the 24 hours before discharge/transfer to *>*50%; and (3) reduce racial/ethnic disparities in mother’s milk use.

**Methods:**

We conducted a quality improvement project from January 2015 to December 2017 focused on prenatal education, first milk expression ≤6 hours after birth, and skin-to-skin care in the first month. We tracked process measures and main outcomes with run and control charts among 202 infants younger than 34 weeks’ gestation eligible to receive mother’s milk born at our hospital; We tracked results according to maternal race/ethnicity subgroups.

**Results:**

Forty-seven percent of mothers were non-Hispanic black, 28% were Hispanic, and 13% were non-Hispanic white. We improved the rate of first milk expression ≤6 hours after birth and skin-to-skin care in the first month but did not improve rates of any/exclusive mother’s milk use at discharge/transfer. Eight-five percent of infants had mothers that initiated milk production, but only 55% received any mother’s milk at discharge/transfer.

**Conclusions:**

Our single-center quality improvement effort focused on infants younger than 34 weeks’ gestation whose mothers were predominately Hispanic and non-Hispanic blacks. We successfully increased first milk expression ≤6 hours after birth and skin-to-skin care but did not increase mother’s milk use at discharge/transfer.

## Abstract 2

Supplemental Digital Content is available in the text.

## INTRODUCTION

Mother’s milk is recommended for preterm infants^1^ because it has numerous health benefits, including a reduction in necrotizing enterocolitis, late-onset sepsis, retinopathy of prematurity, and improved childhood development.^1–3^ However, mothers of preterm infants face significant barriers to on-going milk production, including delayed lactogenesis after delivery, low milk supply, mother-infant separation, and exclusive pumping burden.^4–10^ Hospital practices that support mothers’ milk production include staff and parental education, early milk expression (≤6 hours), and skin-to-skin care.^11–13^

Quality improvement (QI) methodology has been used to increase mother’s milk use for preterm infants through the implementation of hospital breastfeeding support practices.^4,7,8^ Our inner-city hospital, Boston Medical Center (BMC), serves predominantly non-Hispanic black and Hispanic mothers, groups known to have lower breastfeeding rates compared with non-Hispanic white mothers.^14^ At BMC, in early 2015, the rate of mother’s milk initiation among infants <34 weeks’ gestation was >80%, but <60% continued breastfeeding until the point of infant discharge/transfer. Thus, a QI initiative was started, focused on adopting hospital breastfeeding support practices and reducing racial/ethnic disparities in mother’s milk use. The specific aims of our QI initiative, conducted from January 2015 to December 2017, focused on infants younger than 34 weeks, were to (1) increase any mother’s milk use in the 24 hours before discharge/transfer to >75%; (2) increase exclusive mother’s milk use to >50% in the 24 hours before discharge/transfer; and (3) reduce racial/ethnic disparities in mother’s milk use.

## METHODS

### Context

The BMC Neonatal Intensive Care Unit (NICU) is a 22-bed, level III NICU with an average of 2,800 deliveries and 280 NICU admissions annually. BMC received Baby-Friendly designation in 1999, and redesignation in 2007 and 2014. BMC has a donor milk program, established in 2011, to provide donor milk to infants <34 weeks’ gestation when mother’s milk is unavailable.^15^ The BMC NICU has 0.60 FTE dedicated to international board-certified lactation consultants (IBCLC) hours; however, there was a lapse in IBCLC coverage from June 2016 to April 2017. BMC has no NICU lactation peer counselors with personal breastfeeding experience who are trained to help mothers with breastfeeding. Mothers of very low birth weight (VLBW) infants at our hospital routinely receive Medela Symphony pumps after discharge through insurance rental or a hospital loaner program.

**Fig. 1.**
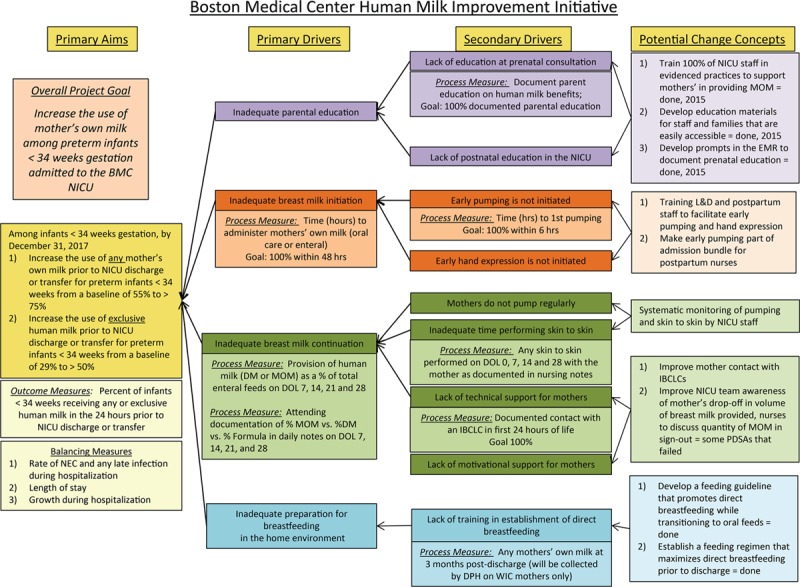
Key driver diagram, created by NeoQIC human milk initiative and adapted for BMC Human Milk Task Force. NeoQIC, Neonatal QI Collaborative of Massachusetts.

We conducted the BMC Mother’s Milk QI initiative within the greater Neonatal QI Collaborative of Massachusetts (NeoQIC) human milk collaborative, a consortium of 10 participating Massachusetts NICUs. NeoQIC leaders provided QI education, data reports, and facilitated team sharing. We adopted the key driver diagram and standard definitions of outcome and process measures from the collaborative with minor modifications specific to our NICU (Fig. 1). Members of our multidisciplinary “Human Milk Task Force” included 1 neonatologist, the NICU nurse manager and nurse educator, 2 medical students, 1 Masters of Public Health student, 3 bedside NICU nurses, 3 IBCLCs, and 1 mother of a NICU patient. This QI project was determined to be non-human subjects research by the institutional review board on our campus.

We tracked data on infants younger than 34 weeks’ gestation born from January 1, 2015, to December 31, 2017. Of 225 infants, we excluded 7 who died before discharge/transfer. One infant was ineligible to receive mother’s milk due to presumed milk-protein allergy, and we excluded 16 infants because their mothers could not provide milk per hospital guidelines. Thus, we included 202 infants in the analysis. Two medical students collected the data from the electronic medical record (EMR). Table 2 includes mother-infant characteristics.

**Table 1.**
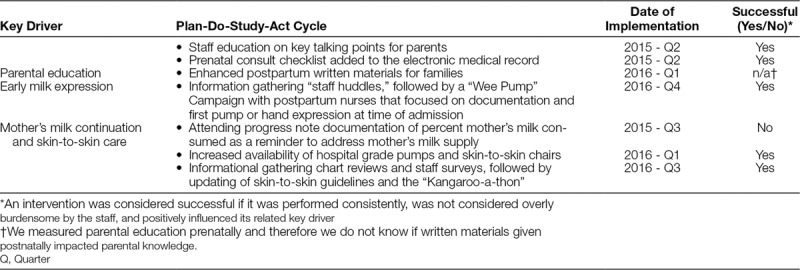
Plan-Do-Study-Act Cycles Implemented by the BMC Human Milk Task Force

#### Interventions.

Several Plan-Do-Study-Act cycles were conducted in line with the Institute for Healthcare Improvement Model for Improvement^16^ (Table 1).

#### Key Driver: Parental Education.

In March–June 2015, the Task Force completed training for all NICU staff, including physicians, nurses, and dieticians. This training focused on the delivery of “talking points” to parents regarding human milk benefits, the need for early and frequent pumping, and the technical training around hand expression and pumping. In May 2015, we implemented a standardized prenatal consultation checklist in the EMR. Physicians documenting a consultation would “check off” if patients’ mothers received human milk education. This standardization reminded providers to deliver education about breastfeeding. In March 2016, we revised written materials delivered to mothers of NICU infants. These materials included information on human milk benefits, pumping and hand expression, choosing the right pump, and sample pumping logs.

#### Key Driver: Early Milk Expression.

To target the early initiation of milk expression <6 hours after birth, we implemented a “Wee Pump” campaign in August 2016 in the postpartum unit. “Huddles” were performed with postpartum nurses to understand their responsibilities and perspectives. We determined that there was confusion about the mode of first milk expression (hand expression versus pump), milk collection supplies, and documenting the time of initiation in the EMR. When beginning the campaign, we clarified these areas of confusion and conducted an education campaign for all postpartum nurses. Then, we provided weekly tracking of time to first milk expression to staff through email and bulletin boards. Nurses who helped mothers achieve the goal of ≤6 hours were publicly congratulated as “Wee Pump” champions, which boosted morale. The nurse manager contacted nurses who did not achieve the goal. Weekly data posting lasted 6 months. We used these practices due to their effectiveness in other NICUs.^17^

#### Key Driver: Mother’s Milk Continuation and Skin-to-Skin Care.

For the first 6 months of 2015, we trialed daily tracking of the percentage of mother’s milk consumed by infants in attending physician progress notes. We hypothesized that a drop-off in the amount of mother’s milk consumed would remind the team to discuss the mother’s milk supply on daily rounds. However, physicians often “cut-and-pasted” inaccurate information, compliance was variable, and it was unclear if recording this in the progress note prompted discussion about the mother’s milk supply during rounds. For these reasons, we stopped this practice.

The task force also focused on the availability of supplies as a barrier to mother’s milk continuation. In March 2016, following the presentation of a business case model to our finance committee, the Task Force acquired new Medela hospital-grade pumps for every postpartum room, NICU bed space, and within the labor and delivery area. Additionally, we wanted to facilitate skin-to-skin care in for our parents to make the experience as enjoyable as possible. In March 2016, the task force used donated funds to purchase 6 large, reclining chairs to facilitate skin-to-skin care.

The Task Force also recognized that staff perceived parental absence as the main barrier to skin-to-skin care. A detailed 2-month retrospective chart review found that parents were present >50% of shifts but performed skin-to-skin care only 25% of the time. This audit indicated that there were missed opportunities for skin-to-skin care, even with parents present. In May 2016, a nursing staff survey revealed major barriers to skin-to-skin care: (1) confusion about eligibility and documentation of skin-to-skin care, (2) completion of clinical duties, and (3) perception of parental desires. Thus, skin-to-skin care guidelines were updated to clarify eligibility, issues with documentation, and identify solutions to nursing workflow barriers. Subsequently, we launched a staff and family awareness campaign (July–August 2016), called the “kangaroo-a-thon.” Skin-to-skin care and parent presence were tracked on each shift, and we shared results weekly accompanied by congratulations to nursing champions. Weekly tracking was stopped after 2 months because data collection was labor-intensive. We continued tracking of skin-to-skin care on specific days per our ongoing data collection.

### Measures

#### Mother’s Milk Metrics/Main Outcomes.

Milk use was abstracted from the medical record on days 7, 14, 21, 28, and in the 24 hours before discharge/transfer as milk received through an NG/OG tube, bottle, or directly at the breast. We defined milk use as either: (1) any mother’s milk, with or without added donor milk, formula, or fortifier; (2) exclusive mother’s milk (100% of the “base” feed was mother’s milk, with or without bovine or human milk fortifier); (3) no mother’s milk (donor milk, formula, or fortifier); or (4) NPO/no feeds. Our main outcome was the percent of infants with any exclusive mother’s milk in the 24 hours before discharge/transfer. All transferred infants were included, regardless of the age at the time of transfer.

#### Process Measures.

Hospital breastfeeding support practices were abstracted from the medical record, and included: (1) prenatal consultation with information about human milk benefits (“human milk,” “breast milk,” “mother’s milk,” “mother’s own milk,” or “breastfeeding,” documented in the prenatal consultation note), among mothers who received a prenatal consultation note (yes/no); (2) initiation of milk expression using pumping/hand expression <6 hours after birth (yes/no); and (3) any skin-to-skin care with the mother performed on days 7, 14, 21, and 28 (yes/no).

#### Other Characteristics.

Other infant characteristics abstracted from the medical record included plurality, birth weight, length, gestational age, and infant gender. Additional outcome measures included the presence of medical or surgical NEC and late-onset bacterial or fungal infection per Vermont Oxford Network definitions.^18^ We categorized maternal race/ethnicity as (1) non-Hispanic white; (2) Hispanic (any race); (3) non-Hispanic black; (4) non-Hispanic Asian; and (5) all others (unknown/declined).

### Analysis

**Fig. 2.**
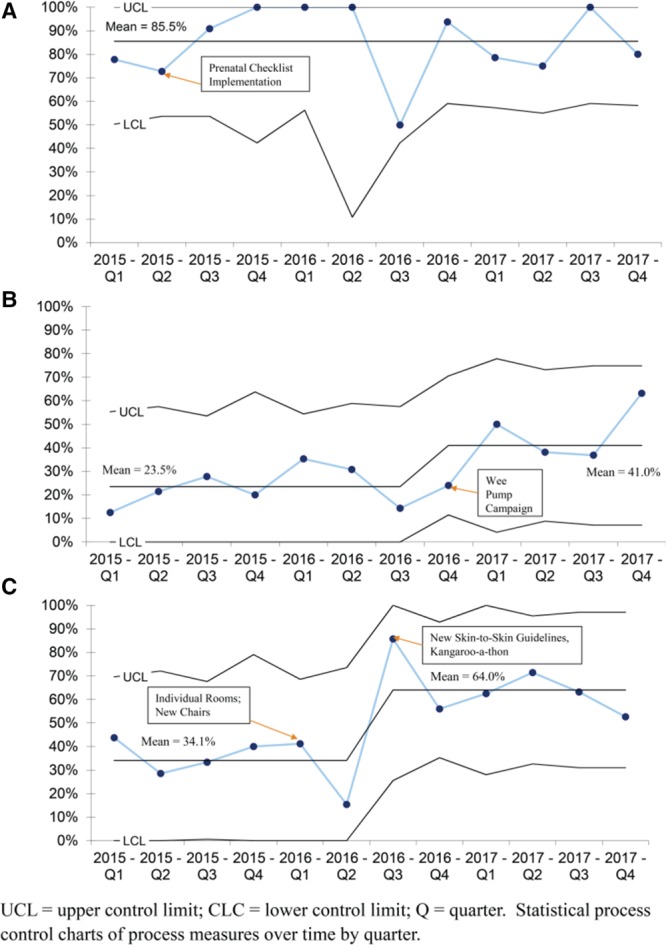
Process measures among infants <34 weeks’ gestation. A, Prenatal consultation include information on human milk benefits, p Chart. B, First milk expression *<*6 hours after birth, p Chart. C, Any skin-to-skin care on day 7, 14, 21, or 28, p Chart.

**Fig. 3.**
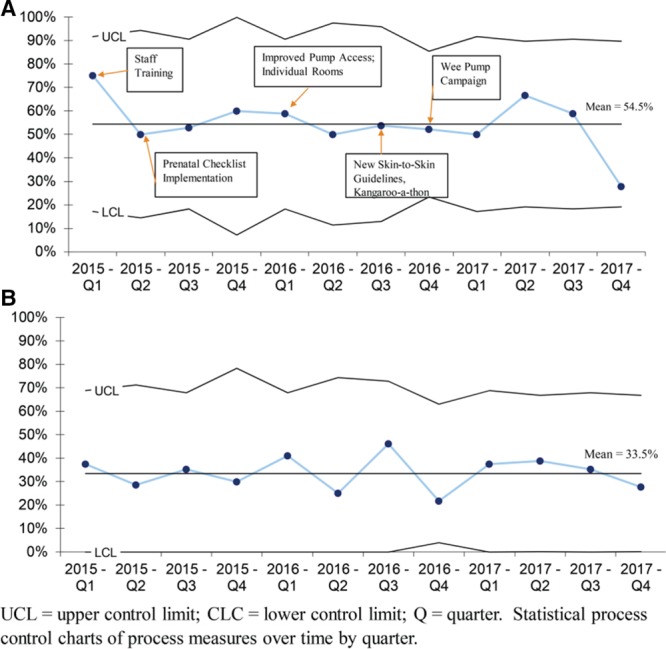
Main outcome measures among infants <34 Weeks’ Gestation. A, *Any* mother’s milk use at discharge/transfer. B, *Exclusive* mother’s milk use at discharge/transfer.

**Fig. 4.**
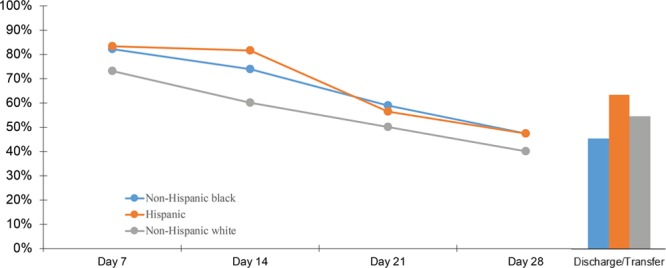
Percent any mother’s milk use during the NICU hospitalization, according to maternal race/ethnicity.

Descriptive characteristics of our cohort were analyzed (Table 2). Statistical process control p-charts were used to examine process measures (Fig. 2) and the main outcome (Fig. 3), by quarter, over time. We analyzed process measures and main outcome by maternal race/ethnicity groups by half-year increments using run charts. [Control charts were not used due to small numbers in each group (see Supplemental Digital Content at http://links.lww.com/PQ9/A124 for Figure 1)]. To understand the timing of cessation in milk production throughout hospitalization, we analyzed the rate of any mother’s milk use on day 7, 14, 21, 28 and at discharge/transfer according to maternal race/ethnicity (Fig. 4). We performed a sub-analysis of our main outcome among VLBW (≤1,500 g) infants (see Supplemental Digital Content at http://links.lww.com/PQ9/A125 for Figure 2). After we found improvement in the process measures focused on the first month of hospitalization, but *not* the main outcome at discharge/transfer, a posthoc p-chart was created to examine changes in any mother’s milk use on day 28. We used Microsoft Excel, with the “QI Charts” add-in.

**Table 2.**
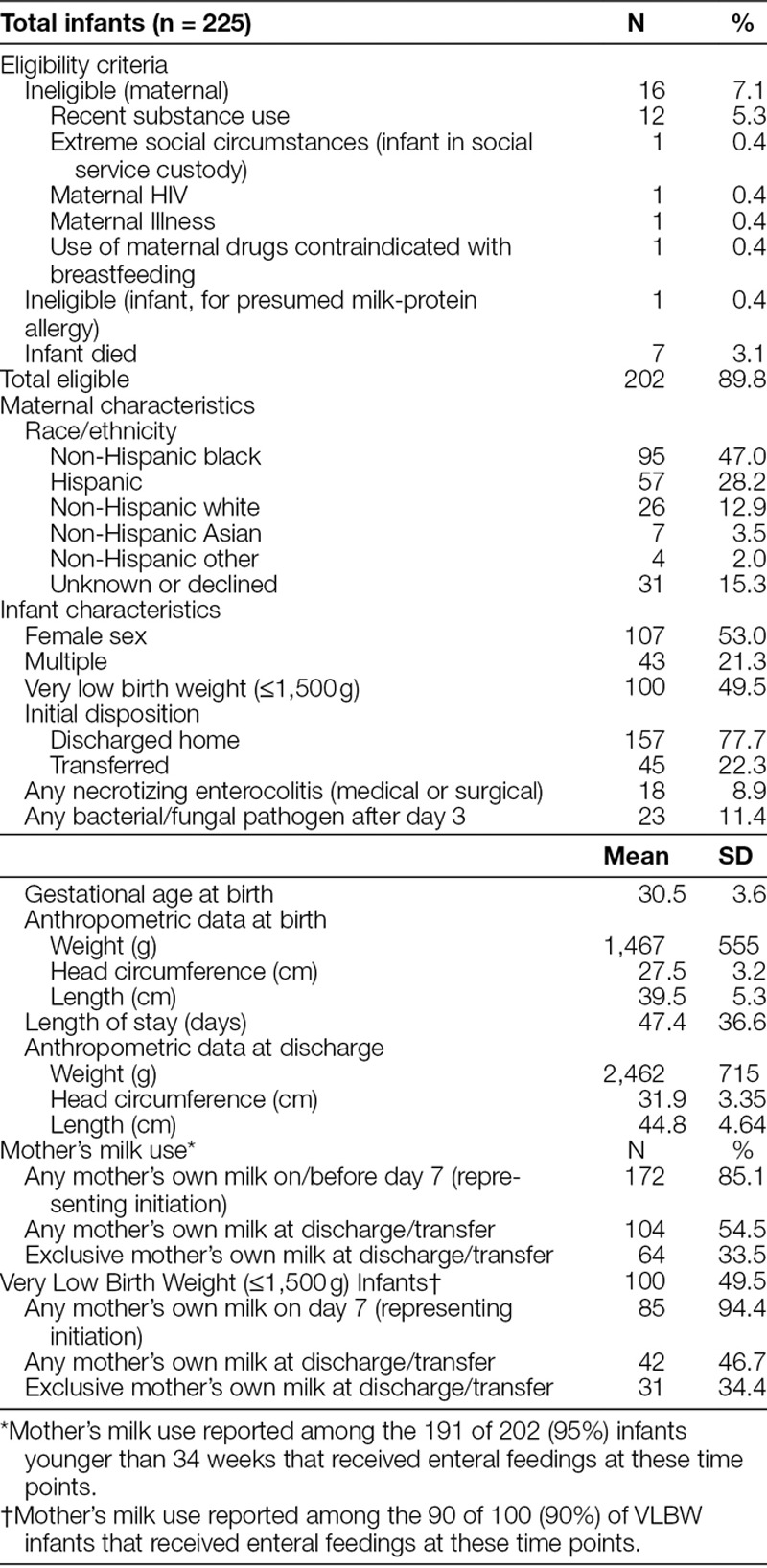
Mother-Infant Characteristics Among Infants Younger Than 34 Weeks’ Gestation Born 01/2015 to 12/2017

## RESULTS

Participant characteristics are shown in Table 2. Of 202 eligible infants, their mothers were 47% non-Hispanic black, 27% Hispanic, and 12% non-Hispanic white. Overall, 85% of mothers initiated making milk for their infants, but 48% had any mother’s milk, and 34% had exclusive use of mother’s milk at discharge/transfer. Among VLBW infants, 94% of mothers initiated making milk, and 47% received any mother’s milk at discharge/transfer.

Regarding our process measures, we found a high rate of human milk education during prenatal consultations (average of 86%) during the QI period (Fig. 2A). First milk expression ≤6 hours after birth improved from an average of 24% before the “Wee Pump” campaign to 41% afterward (Fig. 2B). Skin-to-skin care in the first month improved from an average of 34% before the “Kangaroo-a-thon” to 64% afterward (Fig. 2C). There were no improvements in our main outcomes, any or exclusive mother’s milk at discharge/transfer (Fig. 3). After project completion, we performed a brief chart review on 20 NICU patients in 2019 meeting our eligibility criteria. Of these, 50% had mothers initiate milk expression <6 hours after birth, showing sustained improvement. Skin-to-skin care occurred on 41% of audit days, showing a small sustained improvement.

When process measures (see Supplemental Digital Content at http://links.lww.com/PQ9/A124 for Figure 1) and main outcomes (data not shown) were tracked according to maternal race/ethnicity, we found no notable differences among subgroups. However, since we used half-year increments, there were only 6 data points. Any mother’s milk at discharge/transfer was 45% for infants with non-Hispanic black mothers, 62% for infants with Hispanic mothers, and 54% for infants with non-Hispanic white mothers (*P* = 0.11). Mother’s milk production declined steadily during the NICU hospitalization among all race/ethnicity subgroups (Fig. 4).

In sub-analysis of VLBW infants, we found no changes in any or exclusive mother’s milk use at discharge/transfer (see Supplemental Digital Content at http://links.lww.com/PQ9/A125 for Figure 2). Furthermore, a *posthoc* analysis examining any mother’s milk at day 28, revealed no improvements (data not shown).

## DISCUSSION

Using a QI approach in our inner-city level 3 NICU, early milk expression ≤6 hours after birth and skin-to-skin care in the first month of life improved, but any mother’s milk use at day 28 and at the point of discharge/transfer among infants younger than 34 weeks’ gestation did not. We found no notable differences in the rate of improvement, process measures, or main outcomes according to maternal race/ethnicity. Implementation strategies were well-received by staff and focused on increased awareness, streamlining workflow, and improved access to supplies.

We were surprised that improvements in early milk expression within 6 hours did not yield improvements in mother’s milk use later in hospitalization. Early milk expression within 6 hours has been associated with increased lactation at hospital discharge previously and is recommended by the World Health Organization.^11,19,20^ We speculate that a greater magnitude of improvement in our process measures may have led to longer sustainment of milk production. For example, literature has shown that mothers beginning milk expression within the first hour of delivery is most effective for improving lactation outcomes.^8,11^ While improving expression within 6 hours is an important intervention, it is only one step towards making a meaningful difference in mother’s milk continuation.

Similarly, improvements in skin-to-skin care in the first month did not lead to improvements in mother’s milk use later in the hospitalization. Skin-to-skin care independently has been shown to increase breastfeeding for >6 weeks.^21^ A greater magnitude of improvement or additional focus on skin-to-skin care after a month of life may have led to more sustained improvement.

It is also possible that our focus on only a subset of the factors that impact milk production during NICU hospitalization was too limited. Going forward, we will build on our work to further increase hospital breastfeeding practices in the first month, including the establishment of milk supply, as well as efforts to decrease barriers faced by mothers *after* the first month of life when lactation often ceases. Our improvement in prenatal education likely contributed to the improvement of milk expression <6 hours after birth and the increase in skin-to-skin care in the first month of life, but may require standardized parental education to focus on barriers after this time. We will also focus on the barriers that impact mother-infant separation, such as the cost of parking and restrictions on sibling visitation, and promotion of family support. Our unit would likely benefit from peer lactation counselors, especially given our unique demographics.^10,22^

Work by the Task Force was well-received by staff. Because of positive experiences participating in other NeoQIC initiatives, staff were enthusiastic about participating in NeoQIC’s Human Milk QI Collaborative and were proud of our accomplishments. Participation in the larger collaborative provided access to educational materials for families and education in QI methods, including how to conduct small-scale Plan-Do-Study-Act cycles, and allowed for sharing of ideas with participating hospitals. Additionally, we feel that our project was well-received because our hospital has a long history of breastfeeding promotion.

In our inner-city NICU, comprised primarily of non-Hispanic black and Hispanic mothers, we did not find significant differences in mother’s milk use among racial/ethnic groups at the point of discharge/transfer. This observation differs from published reports, where non-Hispanic blacks and Hispanics have lower rates of mother’s milk use at discharge compared with non-Hispanic whites.^8,23^ Small sample sizes at our hospital may have contributed to the lack of significant differences. Our result may also be in part because our staff are accustomed to addressing communication barriers, social, and economic needs. These factors are barriers to prolonged milk production among many non-Hispanic black and Hispanic mothers.

The rate of initiation of mother’s milk production was 94% in our VLBW infant subgroup, compared with 85% in our larger cohort of infants younger than 34 weeks gestation. We speculate that mothers with VLBW infants perceive their infants as especially vulnerable and are more motivated to produce milk. It is also possible that staff put more emphasis on lactation support for mothers with VLBW infants. Going forward, we will closely consider our strategies to deliver uniform educational messaging and support for mothers of all preterm infants.

Strengths of our study were the use of statistical process control and run charts to analyze changes over time, rather than a pre-post design. We tracked data by maternal race/ethnicity to assess disparities. Limitations include our population and lactation support structure, which may not be generalizable. The changes we implemented may not be useful in all NICU settings. We only tracked skin-to-skin care on specific days, which is less representative than continuous tracking. Additionally, we did not examine infant eligibility for skin-to-skin care, so it is possible that some mothers hoping to perform skin-to-skin care were unable to due to clinical instability. We did not track maternal language, country of origin, breastfeeding intent, pumping frequency or volume, or other socioeconomic factors that are known to impact breastfeeding among mothers of preterm infants.

Notably, the Task Force faced substantial organizational and structural challenges during the QI period. The team lead was on maternity leave for 3 months in 2015; the unit had an interim nurse manager for 9 months in 2015–2016; the lapse of IBCLC coverage from June 2016 to April 2017; and the NICU moved locations in 2016. We recognize the significant work environment contributors to mother’s milk production as well, such as nurse staffing ratios and practice environment that we did not address during this study.^24–26^

## CONCLUSIONS

Using a QI approach, early milk expression and skin-to-skin care were improved in an urban, inner-city level 3 NICU among infants younger than 34 weeks’ gestation. However, mother’s milk use at discharge is affected by more than our early intervention. We achieved success by gathering information through staff interviews and chart reviews, clarifying gaps in workflow processes and staff knowledge, and tracking performance with weekly reports to staff. Enthusiasm for the project and positive feedback were instrumental.

## ACKNOWLEDGMENTS

We acknowledge the members of the BMC Human Milk Task Force, including IBCLCs, dieticians, nurses, and physicians. We acknowledge the families we serve, and Dr. Munish Gupta, NeoQIC director, for his support on this project.

## Supplementary Material
