# Supplementary material for: A Quality Improvement Project to Increase Mother’s Milk Use in an Inner-City NICU
Source: Pediatr Qual Saf. 2019 Aug 30;4(5):e204. doi: 10.1097/pq9.0000000000000204 (PMC6805104; doi:10.1097/pq9.0000000000000204)
Supplement: Supplementary file 1 [file pqs-4-e204-s001.pptx]

## Slide 1
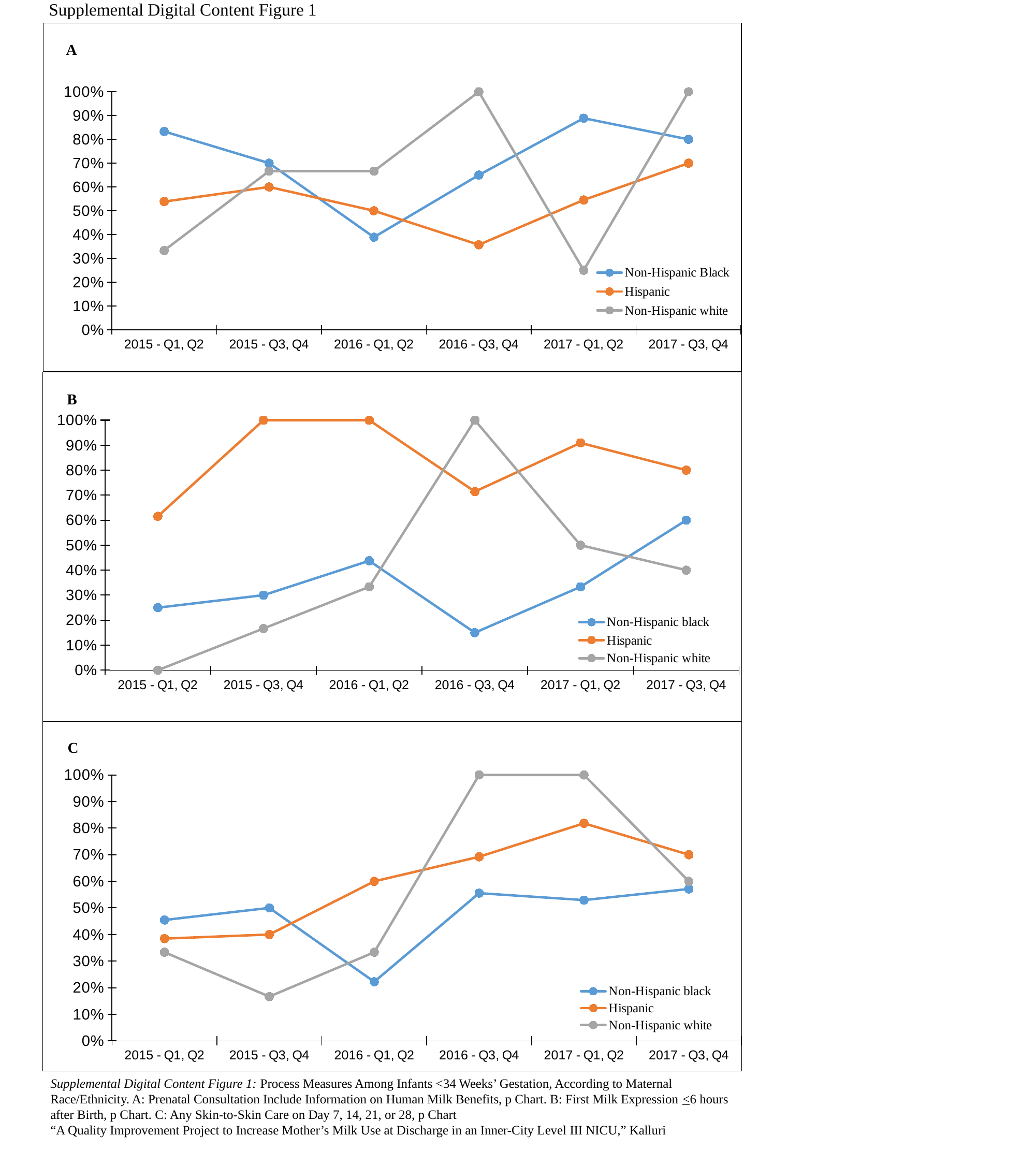

Supplemental Digital Content Figure 1
### Chart: A
| Category | | | |
|---|---|---|---|
| 2015 - Q1, Q2 | 0.8333333333333334 | 0.5384615384615384 | 0.3333333333333333 |
| 2015 - Q3, Q4 | 0.7 | 0.6 | 0.6666666666666666 |
| 2016 - Q1, Q2 | 0.3888888888888889 | 0.5 | 0.6666666666666666 |
| 2016 - Q3, Q4 | 0.65 | 0.35714285714285715 | 1.0 |
| 2017 - Q1, Q2 | 0.8888888888888888 | 0.5454545454545454 | 0.25 |
| 2017 - Q3, Q4 | 0.8 | 0.7 | 1.0 |
### Chart: B
| Category | | | |
|---|---|---|---|
| 2015 - Q1, Q2 | 0.25 | 0.6153846153846154 | 0.0 |
| 2015 - Q3, Q4 | 0.3 | 1.0 | 0.16666666666666666 |
| 2016 - Q1, Q2 | 0.4375 | 1.0 | 0.3333333333333333 |
| 2016 - Q3, Q4 | 0.15 | 0.7142857142857143 | 1.0 |
| 2017 - Q1, Q2 | 0.3333333333333333 | 0.9090909090909091 | 0.5 |
| 2017 - Q3, Q4 | 0.6 | 0.8 | 0.4 |
### Chart: C
| Category | | | |
|---|---|---|---|
| 2015 - Q1, Q2 | 0.45454545454545453 | 0.38461538461538464 | 0.3333333333333333 |
| 2015 - Q3, Q4 | 0.5 | 0.4 | 0.16666666666666666 |
| 2016 - Q1, Q2 | 0.2222222222222222 | 0.6 | 0.3333333333333333 |
| 2016 - Q3, Q4 | 0.5555555555555556 | 0.6923076923076923 | 1.0 |
| 2017 - Q1, Q2 | 0.5294117647058824 | 0.8181818181818182 | 1.0 |
| 2017 - Q3, Q4 | 0.5714285714285714 | 0.7 | 0.6 |Supplemental Digital Content Figure 1: Process Measures Among Infants <34 Weeks’ Gestation, According to Maternal Race/Ethnicity. A: Prenatal Consultation Include Information on Human Milk Benefits, p Chart. B: First Milk Expression <6 hours after Birth, p Chart. C: Any Skin-to-Skin Care on Day 7, 14, 21, or 28, p Chart
“A Quality Improvement Project to Increase Mother’s Milk Use at Discharge in an Inner-City Level III NICU,” Kalluri
