# Supplementary material for: A Quality Improvement Project to Increase Mother’s Milk Use in an Inner-City NICU
Source: Pediatr Qual Saf. 2019 Aug 30;4(5):e204. doi: 10.1097/pq9.0000000000000204 (PMC6805104; doi:10.1097/pq9.0000000000000204)
Supplement: Supplementary file 2 [file pqs-4-e204-s002.pptx]

## Slide 1
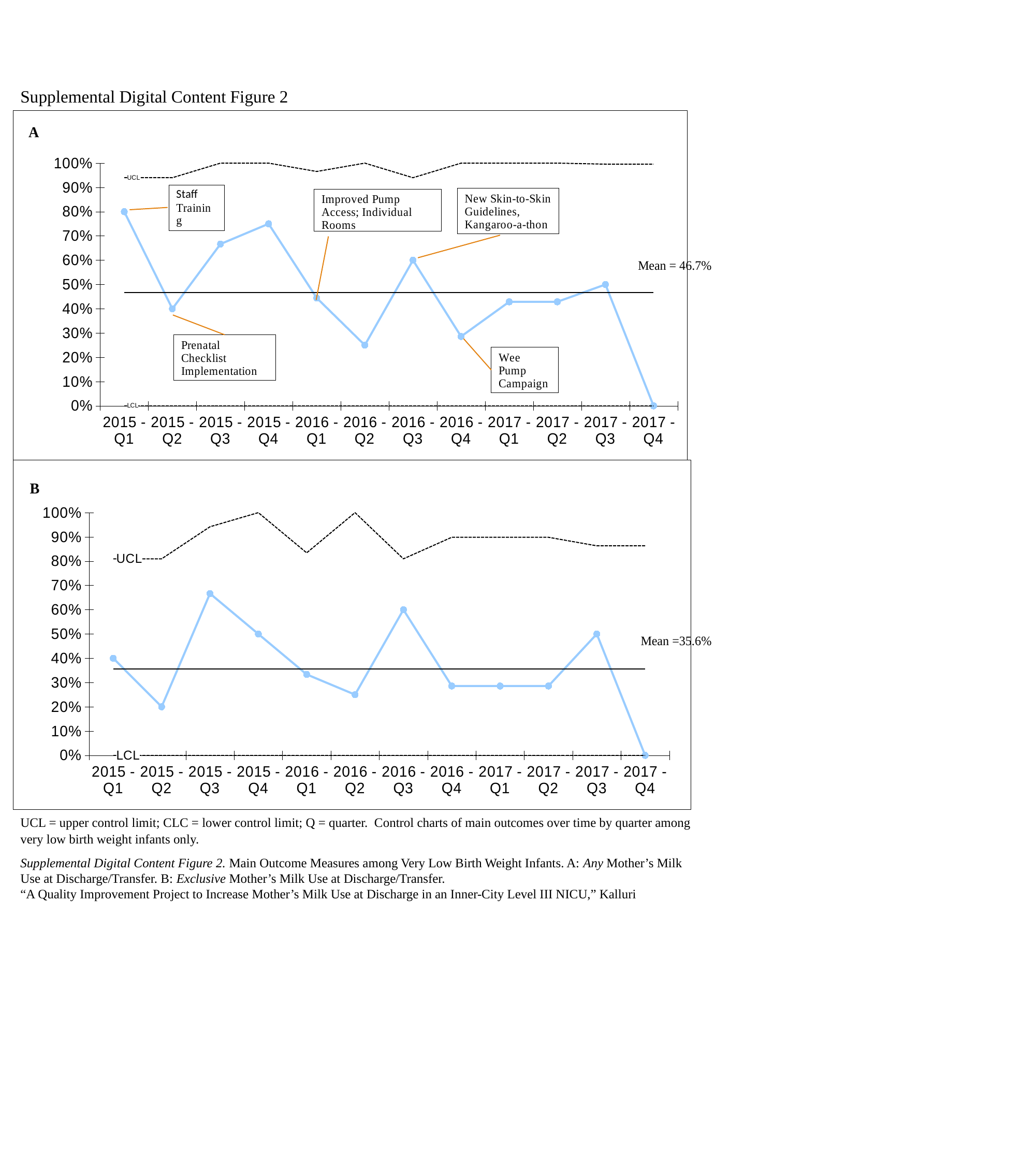

Supplemental Digital Content Figure 2
### Chart: A
| Category | | | | |
|---|---|---|---|---|
| 2015 - Q1 | 0.8 | 0.4666666666666667 | 0.939953049314636 | 0.0 |
| 2015 - Q2 | 0.4 | 0.4666666666666667 | 0.939953049314636 | 0.0 |
| 2015 - Q3 | 0.6666666666666666 | 0.4666666666666667 | 1.0 | 0.0 |
| 2015 - Q4 | 0.75 | 0.4666666666666667 | 1.0 | 0.0 |
| 2016 - Q1 | 0.4444444444444444 | 0.4666666666666667 | 0.9655543182365255 | 0.0 |
| 2016 - Q2 | 0.25 | 0.4666666666666667 | 1.0 | 0.0 |
| 2016 - Q3 | 0.6 | 0.4666666666666667 | 0.939953049314636 | 0.0 |
| 2016 - Q4 | 0.2857142857142857 | 0.4666666666666667 | 1.0 | 0.0 |
| 2017 - Q1 | 0.42857142857142855 | 0.4666666666666667 | 1.0 | 0.0 |
| 2017 - Q2 | 0.42857142857142855 | 0.4666666666666667 | 1.0 | 0.0 |
| 2017 - Q3 | 0.5 | 0.4666666666666667 | 0.9958169288795848 | 0.0 |
| 2017 - Q4 | 0.0 | 0.4666666666666667 | 0.9958169288795848 | 0.0 |
### Chart: B
| Category | | | | |
|---|---|---|---|---|
| 2015 - Q1 | 0.4 | 0.35555555555555557 | 0.809672525313593 | 0.0 |
| 2015 - Q2 | 0.2 | 0.35555555555555557 | 0.809672525313593 | 0.0 |
| 2015 - Q3 | 0.6666666666666666 | 0.35555555555555557 | 0.9418180425899074 | 0.0 |
| 2015 - Q4 | 0.5 | 0.35555555555555557 | 1.0 | 0.0 |
| 2016 - Q1 | 0.3333333333333333 | 0.35555555555555557 | 0.8342368717452893 | 0.0 |
| 2016 - Q2 | 0.25 | 0.35555555555555557 | 1.0 | 0.0 |
| 2016 - Q3 | 0.6 | 0.35555555555555557 | 0.809672525313593 | 0.0 |
| 2016 - Q4 | 0.2857142857142857 | 0.35555555555555557 | 0.8983291497946035 | 0.0 |
| 2017 - Q1 | 0.2857142857142857 | 0.35555555555555557 | 0.8983291497946035 | 0.0 |
| 2017 - Q2 | 0.2857142857142857 | 0.35555555555555557 | 0.8983291497946035 | 0.0 |
| 2017 - Q3 | 0.5 | 0.35555555555555557 | 0.8632737626131495 | 0.0 |
| 2017 - Q4 | 0.0 | 0.35555555555555557 | 0.8632737626131495 | 0.0 |UCL = upper control limit; CLC = lower control limit; Q = quarter. Control charts of main outcomes over time by quarter among very low birth weight infants only.
Supplemental Digital Content Figure 2. Main Outcome Measures among Very Low Birth Weight Infants. A: Any Mother’s Milk Use at Discharge/Transfer. B: Exclusive Mother’s Milk Use at Discharge/Transfer.
“A Quality Improvement Project to Increase Mother’s Milk Use at Discharge in an Inner-City Level III NICU,” Kalluri
